# Evolution of gut *Bifidobacterium* population in healthy Japanese infants over the first three years of life: a quantitative assessment

**DOI:** 10.1038/s41598-017-10711-5

**Published:** 2017-08-30

**Authors:** Ravinder Nagpal, Takashi Kurakawa, Hirokazu Tsuji, Takuya Takahashi, Kazunari Kawashima, Satoru Nagata, Koji Nomoto, Yuichiro Yamashiro

**Affiliations:** 1 Probiotics Research Laboratory, Juntendo University Graduate School of Medicine, Hongo 2-9-8-3F, Bunkyo-ku, Tokyo 113-0033 Japan; 2 Yakult Central Institute, Kunitachi-shi, Tokyo Japan; 3 Gonohashi Obstetrics and Gynecology Hospital, Koto-ku, Tokyo Japan; 4 Department of Pediatrics, School of Medicine, Tokyo Women’s Medical University, Shinjuku, Tokyo Japan; 5 Present Address: Gut Microbiome and Metabolic Diseases, Center for Diabetes, Obesity and Metabolism, Wake Forest School of Medicine, Biotech Place, Winston-Salem, NC 27101 USA

## Abstract

Bifidobacteria are important members of human gut microbiota; however, quantitative data on their early-life dynamics is limited. Here, using a sensitive reverse transcription-qPCR approach, we demonstrate the carriage of eight signature infant-associated *Bifidobacterium* species (*B. longum, B. breve, B. bifidum, B. catenulatum* group*, B. infantis, B. adolescentis, B. angulatum* and *B. dentium*) in 76 healthy full-term vaginally-born infants from first day to three years of life. About 21% babies carry bifidobacteria at first day of life (6.2 ± 1.9 log_10_ cells/g feces); and this carriage increases to 64% (8.0 ± 2.2), 79% (8.5 ± 2.1), 97% (9.3 ± 1.8), 99% (9.6 ± 1.6), and 100% (9.7 ± 0.9) at age 7 days, 1, 3 and 6 months, and 3 years, respectively. *B. longum, B. breve, B. catenulatum* group and *B. bifidum* are among the earliest and abundant bifidobacterial clades. Interestingly, infants starting formula-feed as early as first week of life have higher bifidobacterial carriage compared to exclusively breast-fed counterparts. Bifidobacteria demonstrate an antagonistic correlation with enterobacteria and enterococci. Further analyses also reveal a relatively lower/ delayed bifidobacterial carriage in cesarean-born babies. The study presents a quantitative perspective of the early-life gut *Bifidobacterium* colonization and shows how factors such as birth and feeding modes could influence this acquisition even in healthy infants.

## Introduction

Bifidobacteria represent one of the earliest and most abundant bacterial colonizers of the neonatal gut and are well known to confer a myriad of benefits to the host intestinal, metabolic and immune health^1–7^. The genus *Bifidobacterium* belongs to the family Bifidobacteriaceae, order Bifidobacteriales and phylum Actinobacteria; and comprises a gut commensal clade of Gram-positive, polymorphic rod-shaped, high G + C bacteria that constitutes almost 10% of the typical human adult intestinal microbiota^4, 8^. While the genus *Bifidobacterium* is comprised of more than 50 species/subspecies, the typical human-associated species include *B. bifidum, B. breve*, *B. longum, B. infantis, B. catenulatum, B. pseudocatenulatum*, *B. adolescentis*, *B. angulatum, B. dentium*, and *B. pseudolongum* ^4, 5, 7, 9, 10^. However, despite their perceived significance, the precise succession ontogeny of these bifidobacterial species during infancy and early childhood remains to be comprehended, particularly in terms of their actual population levels i.e., the absolute bacterial count. Most of the studies have performed bifidobacterial enumeration either on a single time-point or in relatively smaller cohorts which may not yield clear-cut information since the microbiota composition, though relatively simple, remains fluctuating during early years of life. Further, majority of longitudinal studies have used bacterial rRNA gene-based PCR or sequencing methods^2, 5, 6, 11–13^. No doubt the sequencing methods are elegantly elaborative and comprehensive, but these are largely qualitative and do not provide numerical data of bacterial counts. Also, sequence resolution may not be sufficient for species-level analyses; for example, bifidobacterial species remain under-resolved in sequence datasets generated from 16 S V3/V4 regions as compared to 16 S V1-V2 sequences which provide adequate sequence variation for species-level discrimination of bifidobacteria^7^. In addition, the data generated from DNA-based methods may include background DNA (e.g., free bacterial DNA, or DNA of dead bacteria) present in the feces and hence may not truly distinguish between viable and dead bacteria. As a result, the numerical data about the intestinal carriage of bifidobacterial community in infants remains limited and disparate. Furthermore, various postnatal elements such as birth mode and feeding type can have strong impact on infant microbiota^1, 5, 6, 14–16^, thereby underscoring the importance of assessing the bifidobacterial microbiota in larger cohorts. Notably, most formula-feed supplements presently prevalent in Japan are fortified with different prebiotics such as galacto-oligosaccharides, fructo-oligosaccharides, lactulose, raffinose etc.; however, the data on the influence of these compounds on the fecal bifidobacterial levels during infancy remain limited.

In these contexts, we herein aimed to define the quantitative profile of gut bifidobacterial microbiota during early life in a relatively large cohort of 76 healthy full-term vaginally-born Japanese infants by employing a sensitive analytical approach based on reverse transcription-quantitative-PCR (RT-qPCR) targeting bacterial 16 S rRNA molecules. The method is relatively highly sensitive (detection limit: 10^4^ cells/g feces), which is particularly important for analysis during neonatal stages since the microbiota is underdeveloped. Another advantage is that this method provides information on viable cells^17–22^. In our previous methodological studies, we have validated that the counts obtained by this RT-qPCR system are equivalent to the bacterial counts obtained by culture and fluorescent *in-situ* hybridization (FISH) methods irrespective of the bacterial growth phase^17, 18^, and that the analytical sensitivity of RT-qPCR is approximately 100- to 1000-fold higher than that of other molecular methods including qPCR and T-RFLP^17, 18, 20, 22^. Therefore, owing to these advantages, we specifically employed RT-qPCR analysis for the present study. Herein we demonstrate a quantitative perspective of the age-related dynamics of typical infant-associated *Bifidobacterium* species in infant gut prospectively from birth to 3 years of age, in particular context to the colonization patterns, birth mode, feeding type, and correlation with other gut bacteria. The data provide important numerical information about the population levels (i.e. the actual bacterial count) of human gut bifidobacterial community during infancy and early childhood and hence shall be informative and facilitative for prospective microbiota-related studies.

## Results

### A quantitative perspective of the succession of gut *Bifidobacterium* microbiota during early life

**Figure 1:**
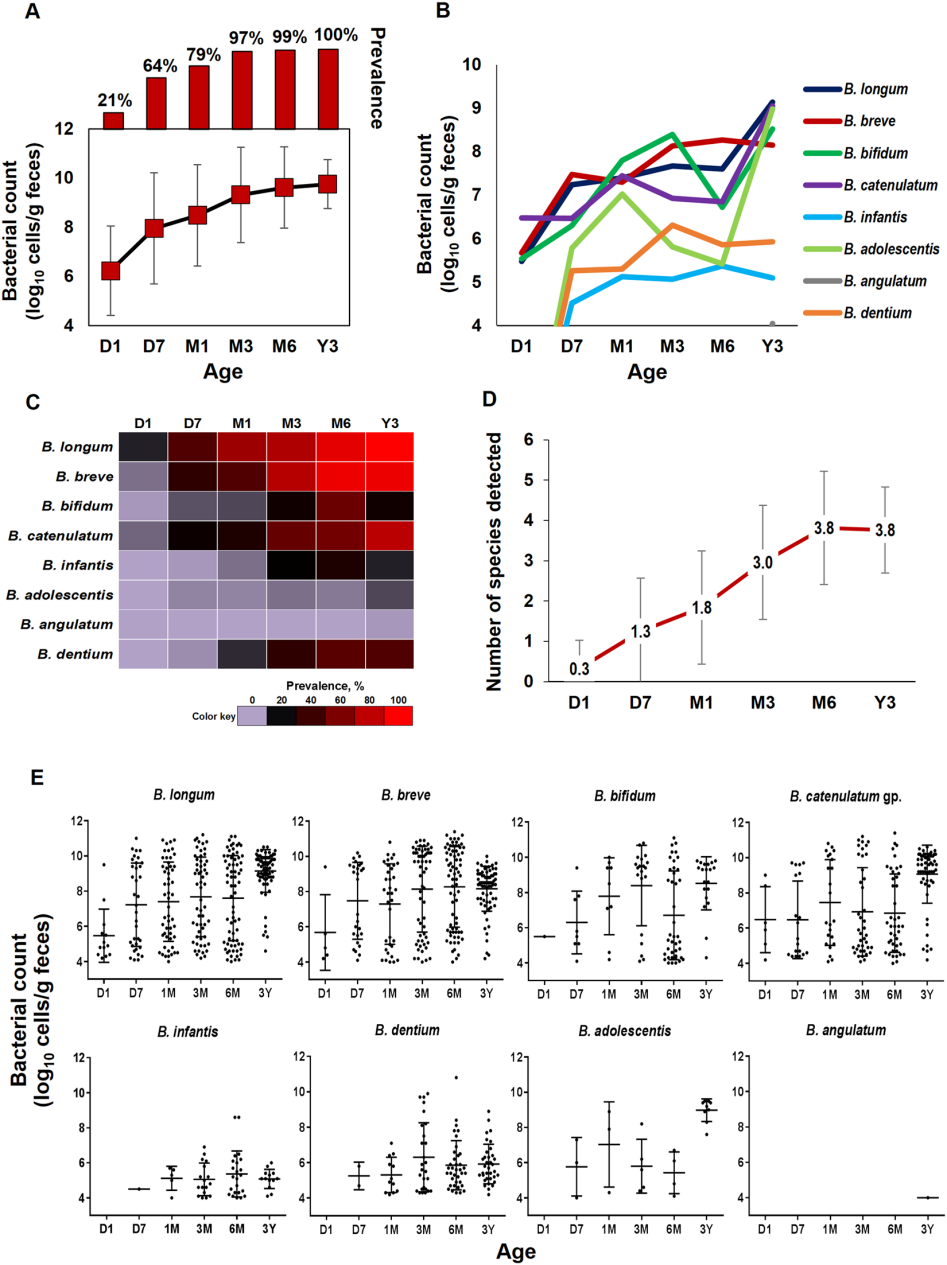
Dynamics of the fecal *Bifidobacterium* carriage during the first 3 years of life. (**1A**) Fecal count and prevalence of genus *Bifidobacterium* at different time-points. (**1B**) Fecal counts of *Bifidobacterium* species. (**1C**) Heat-map showing the prevalence of *Bifidobacterium* species. (**1D**) Average number (mean ± SD) of *Bifidobacterium* group/ species detected at each time-point. (**1E**) Scatter dot-plots showing the fecal counts of *Bifidobacterium* species at different time-points (horizontal lines and error bars represent the mean and standard deviation, respectively). Bacterial count (log_10_ cells/g feces) is expressed as mean ± standard deviation. Prevalence (detection rate, %) is expressed as the percentage of infants in which the specific bacterium was detected. The count of genus *Bifidobacterium* represents the sum of the fecal counts of *B. longum* subsp*. longum, B. longum* subsp*. infantis, B. breve, B. catenulatum* group*, B. bifidum, B. adolescentis, B. angulatum* and *B. dentium*. *B. catenulatum* group includes *B. catenulatum* and *B. pseudocatenulatum*. Age (*x*-axis): 1 day, 7 days, 1 month, 3 months, 6 months, 3 years.

As depicted in Fig. 1A, 21% of babies carried bifidobacteria at first day of life i.e., in the first meconium sample (mean *Bifidobacterium* count: 6.2 ± 1.9 log_10_ cells/g feces), and this prevalence (and count) gradually increased to 64% (8.0 ± 2.2), 79% (8.5 ± 2.1), 97% (9.3 ± 1.8), and 99% (9.6 ± 1.6) at age 7 days, 1, 3 and 6 months, respectively. At age 3 years, all babies carried bifidobacteria (mean *Bifidobacterium* count: 9.7 ± 0.9 log_10_ cells/g). The count and prevalence of individual bifidobacterial groups and species are presented in Fig. 1B and C, respectively. *B. longum* subsp. *longum (*hereafter *B. longum), B. breve, B. catenulatum* group and *B. bifidum* were the first colonizers (detected at day 1) and followed similar trend of fecal carriage as that of genus *Bifidobacterium* during subsequent time-points. *B. longum* subsp*. infantis* (hereafter *B. infantis*)*, B. dentium* and *B. adolescentis* appeared at day 7 whereas *B. angulatum* was detected only at 3 years. In terms of count as well as prevalence, *B. longum, B. breve*, and *B. catenulatum* group remained dominant bifidobacterial clades throughout the study period. The number of bifidobacterial species detected at a given time-point is presented in Fig. 1D. On average, the infants in this cohort carried 2 or less species at D1 (average number of species detected: 0.3 ± 0.7), but this carriage rate gradually increased on subsequent time-points with as many as 5 to 7 species detected at D7 (1.3 ± 1.3), M1 (1.8 ± 1.4), M3 (3.0 ± 1.4), M6 (3.8 ± 1.4), and Y3 (3.8 ± 1.1). As shown in Fig. 1E, the scattered dot-plots of individual bifidobacterial group and species also indicated a gradual increase in the bifidobacterial diversity, while showing that the intestinal carriage of bifidobacteria remains widely different during the first 6 months but reaches a relatively stable state by age 3 years (Fig. 1E).

### Infant nutrition affects gut *Bifidobacterium* carriage during early childhood

**Figure 2:**
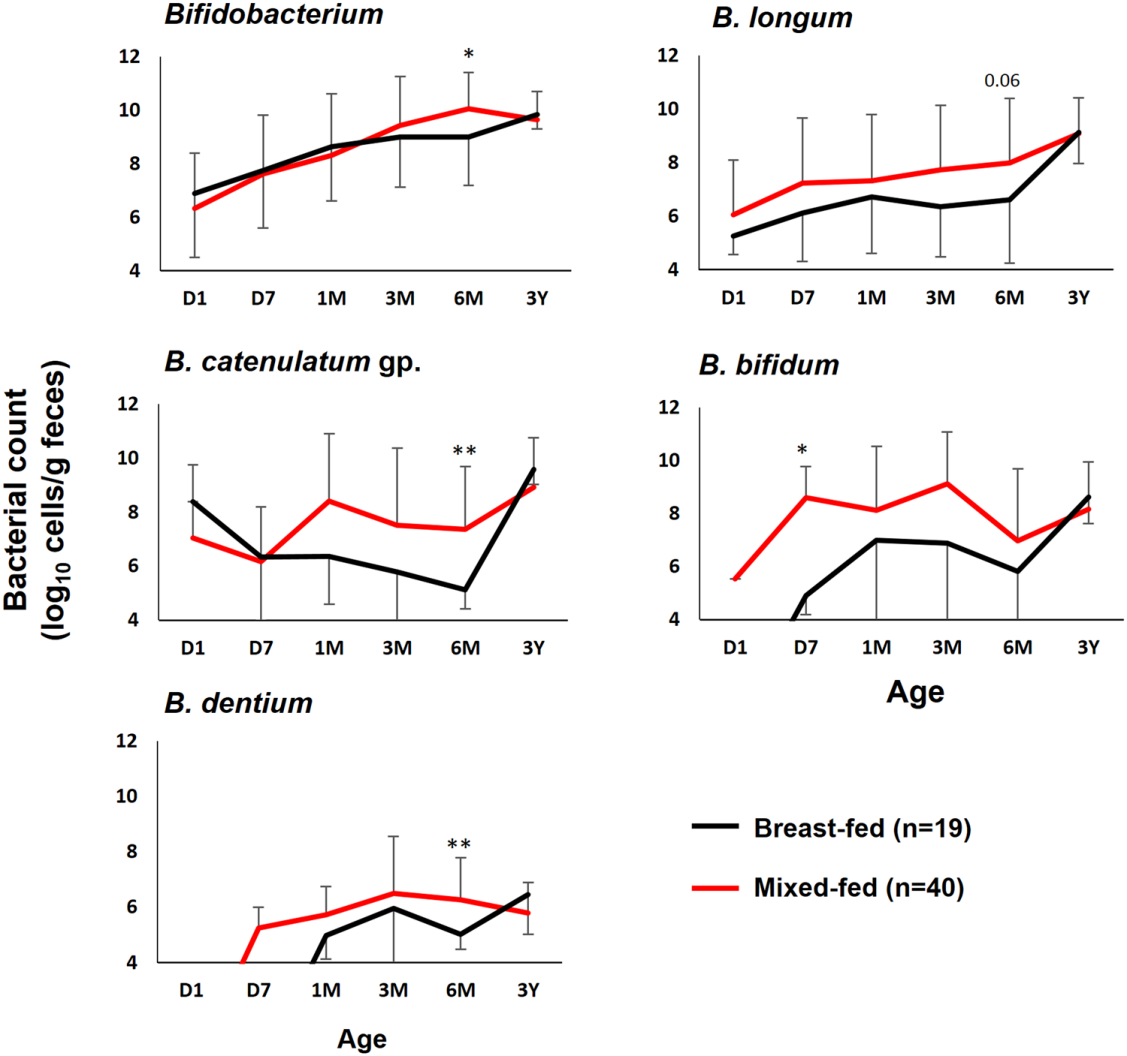
Infant nutrition affects gut *Bifidobacterium* carriage during early childhood. Fecal counts of bifidobacteria in vaginally-born babies that started receiving formula-feed at age 7 days (mixed-fed) vs. those who remained exclusively breast-fed during the first 3 months of life (breast-fed). Bacterial count (log_10_ cells/g feces) is expressed as mean ± SD. The count of genus *Bifidobacterium* is expressed as the sum of the counts (log_10_ cells/g feces) of *B. longum* subsp*. longum, B. longum* subsp*. infantis, B. breve, B. catenulatum* group*, B. bifidum, B. adolescentis, B. angulatum* and *B. dentium*. *B. catenulatum* group includes *B. catenulatum* and *B. pseudocatenulatum*. *P < 0.05, **P < 0.01 (Student’s *t*-test). Only bacteria with notable difference are shown here. Age (*x*-axis): 1 day, 7 days, 1 month, 3 months, 6 months, 3 years.

To analyze the effect of feeding, the data of infants with a consistent mode of feeding during the first three months (i.e., either breast-fed or mixed-fed unvaryingly from D7 to M3) were divided into two groups: (i) infants exclusively breast-fed from birth to M3 (BF; n = 19), and (ii) infants receiving formula-feed from D7 to M3 (MF; n = 40). Data of remaining 17 infants was excluded from feeding analysis because of inconsistent feeding regimen during the first three months. At D1, all babies were breast-fed; or in other words, none of the baby received any form of formula feed or supplement until the collection of first sample. The results of bacterial counts by the mode of feeding are presented in Fig. 2. Babies starting formula-feed supplementation as early as first week of life (MF) had higher carriage of all major bifidobacterial members at one or more time-points during the first 6 months of life as compared to babies exclusively breast-fed during first 3 months (BF). However, as in the case of birth mode, all these differences disappeared by 3 years of age. In terms of prevalence, no significant difference was observed between the two feeding groups.

### Bifidobacteria share wide-ranging correlation arrays with other gut bacteria

**Figure 3:**
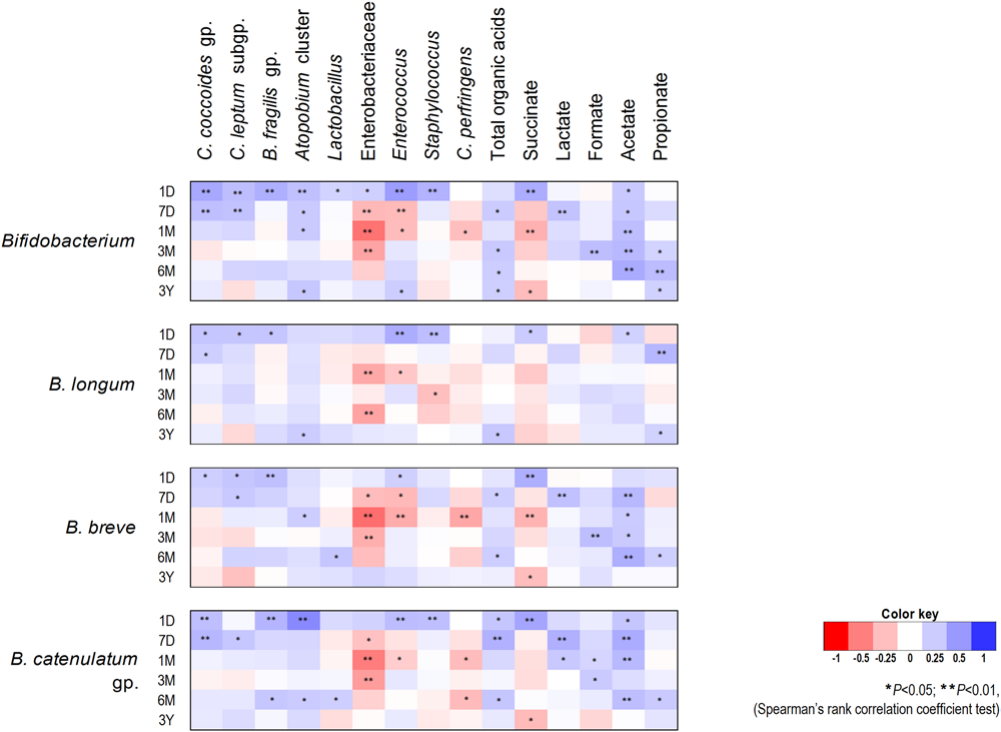
Bifidobacteria exhibit wide-ranging correlation arrays with other gut bacterial clades and fecal organic acids. Heat-map depicting the numerical correlation of major bifidobacterial clades with other gut bacteria (log_10_ cells/g feces) and organic acids (µmol/g feces) in healthy full-term vaginally-born infants (n = 76) at different time-points during the first 3 years of life. The count of genus *Bifidobacterium* is expressed as the sum of the counts of *B. longum* subsp*. longum, B. longum* subsp*. infantis, B. breve, B. catenulatum* group*, B. bifidum, B. adolescentis, B. angulatum* and *B. dentium*. *B. catenulatum* group includes *B. catenulatum* and *B. pseudocatenulatum*. Spearman’s rank correlation coefficients is indicated by color gradient: red denotes negative correlation; Blue denotes positive correlation. *P < 0.05, **P < 0.01. Age (*y*-axis): 1 day, 7 days, 1 month, 3 months, 6 months, 3 years.

To estimate the relationship of bifidobacteria with other bacteria dwelling in the gut of these 76 babies, we performed numerical correlation analysis of bifidobacterial counts against the counts of other major gut bacterial clades viz. *Clostridium coccoides* group, *Clostridium leptum* subgroup, *Bacteroides fragilis* group, *Atopobium* cluster, *Prevotella*, *Lactobacillus*, Enterobacteriaceae, *Enterococcus*, *Staphylococcus, Streptococcus*, and *C. perfringens*. In addition, correlation assessment was also done for fecal concentration of organic acids including acetate, lactate, succinate, propionate, butyrate, and valerate. The data of gut bacteria (log_10_ cells/g feces, as enumerated by same RT-qPCR based approach) and organic acids (µmol/g feces) were retrieved from a preceding project, as reported previously^23^. The results of correlation analyses are presented in Fig. 3. All the bifidobacterial members appeared to exhibit a similar pattern of correlation with other gut bacteria and organic acids (Fig. 3), with a significant (*P* < 0.05 or *P* < 0.01) positive correlation with *C. coccoides* group, *C. leptum* subgroup, *B. fragilis* group, *Atopobium* cluster, and *Staphylococcus* and a significant (*P* < 0.05 or *P* < 0.01) negative correlation with Enterobacteriaceae, *Enterococcus* and *C. perfringens* consistently or inconsistently at one or more time-points during the first 6 months. Because all bifidobacterial clades displayed similar correlation patterns, the data of only major clades is shown in here. Notably, the correlation was most prominent in the case of Enterobacteriaceae. As expected, bifidobacteria shared a positive correlation with fecal organic acids with correlation being most prominent for acetic acid. However, the correlation was found to be negative in the case of succinic acid at one or more time-points (Fig. 3).

### Birth mode may influence early-life gut *Bifidobacterium* carriage

**Figure 4:**
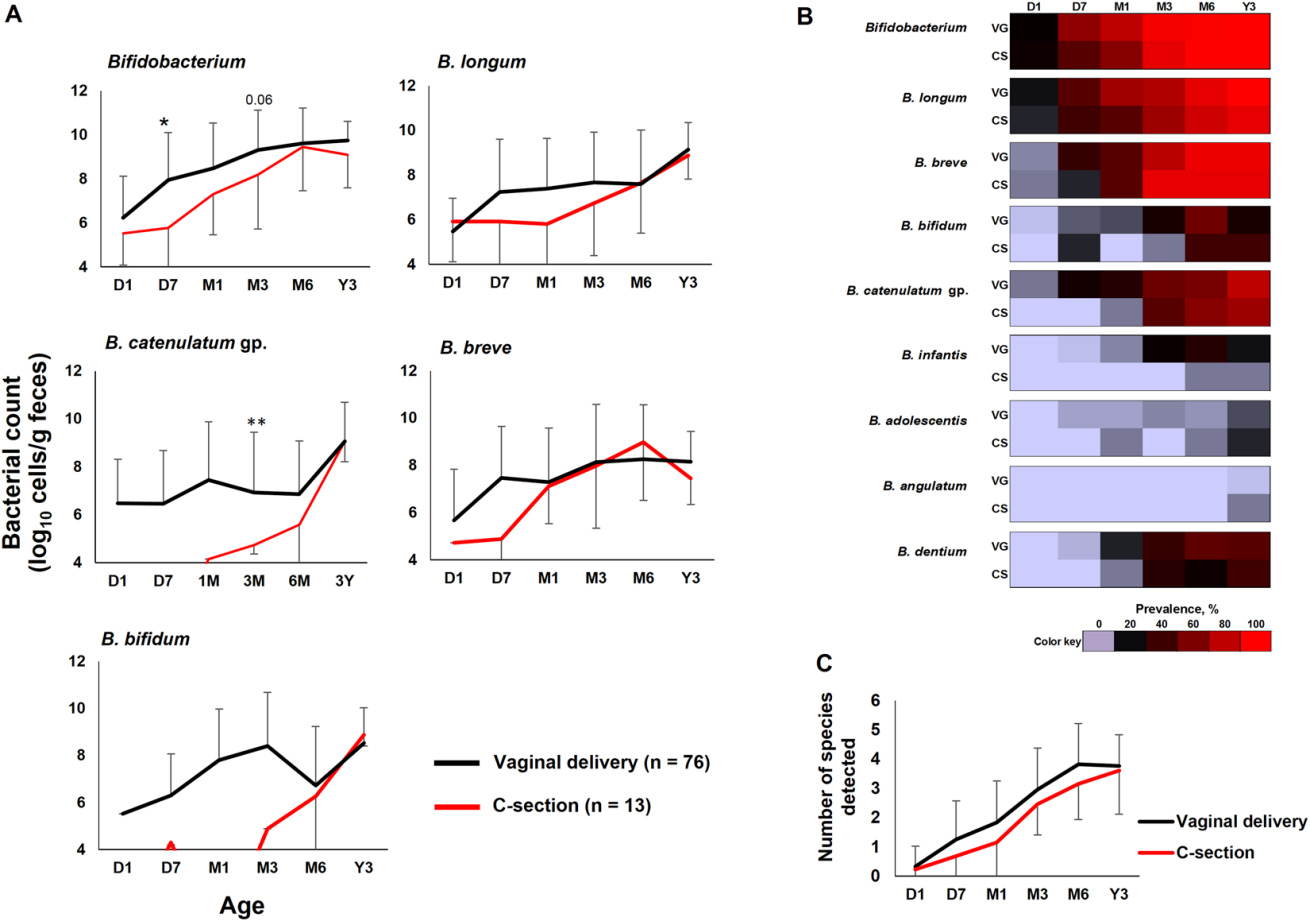
Birth mode may influence early-life gut *Bifidobacterium* carriage. Differences in the fecal count (**4A**) and prevalence (**4B**) of bifidobacterial species, and average (mean ± SD) number of species detected (**4C**) between vaginally- and cesarean-born babies. Bacterial count (log_10_ cells/g feces) is expressed as mean ± SD. Prevalence (detection rate, %) is expressed as the percentage of infants in which the specific bacterium was detected. The count of genus *Bifidobacterium* represents the sum of the counts of *B. longum* subsp*. longum, B. longum* subsp*. infantis, B. breve, B. catenulatum* group*, B. bifidum, B. adolescentis, B. angulatum* and *B. dentium*. *B. catenulatum* group includes *B. catenulatum* and *B. pseudocatenulatum*. Age (*x*-axis): 1 day, 7 days, 1 month, 3 months, 6 months, 3 years. VG: vaginal delivery; CS: C-section. *P < 0.05, **P < 0.01 (Student’s *t*-test). Only bacteria with notable difference are shown in Fig. 4A. Age (*x*-axis): 1 day, 7 days, 1 month, 3 months, 6 months, 3 years.

To estimate the effect of birth mode on early-life bifidobacterial carriage, we compared the data of these 76 vaginally-born (VG) infants with a small counterpart group of 13 babies that were delivered via elective C-section (CS). These results are presented in Fig. 4. Interestingly, compared to VG infants, CS infants had considerably lower count of major bifidobacterial clades at one or more time-points during the first 3 months (Fig. 4A). In addition, as depicted in the form of a heat-map in Fig. 4B, CS infants also appeared to exhibit slightly lower or delayed carriage rate of several bifidobacterial clades, mainly *B. catenulatum* group, *B. dentium* and *B. infantis*, during the first 6 months. In line with this, the average of number of species detected at different time-points during the first 6 months remained on a higher side in VG infants compared to CS infants (Fig. 4C). However, all these differences tended to diminish by the age of 3 years. Because infants acquired bifidobacteria at specific time-points, this also allowed us to arbitrarily define three types of bifidobacterial colonization patterns in this cohort: early (from D1 to D7), intermediate (at M1) and late (M3 or later) colonization. This aggregated colonization pattern of *Bifidobacterium* in 89 infants is presented in the form of a clustered heat-map in Suppl. Fig. 1. About 63% of babies (n = 56) were found to be early-carriers, 21% were intermediate colonizers (n = 19), while rest 16% of the babies (n = 14) were late-carriers. Notably, from the point of first detection, the colonization remained mostly continuous during all subsequent time-points. As such, there was no clear correlation of these patterns with birth mode, but we noted that most of the CS babies were in either intermediate or late colonization group (Suppl. Fig. 1).

## Discussion

To our knowledge, this appears to be the largest longitudinal study quantifying the gut *Bifidobacterium* carriage in healthy full-term infants from birth to 3 years of age and analyzing the influence of birth and feeding modes on its succession. To quantify fecal bifidobacteria, we herein used 16 S rRNA molecules-targeted RT-qPCR mainly because its competent analytical sensitivity (10^4^ cells/g feces) and advantage of providing information about the viable cells^17–22^. Bifidobacteria may sometimes remain under-represented or under-estimated in 16 S rRNA gene sequencing-based datasets (particularly during early life when these are not essentially dominant or abundant at all times)^11, 24^ because of technical glitches such as low detection sensitivity, the biasness of particular PCR primer against 16 S rRNA gene sequences, or the use of DNA extraction methods that are not adequately compatible for (high G + C, Gram-positive) bifidobacteria^25, 26^. Also, sequencing methods generate tens of thousands of rRNA gene sequences per DNA sample but there might be several hundred OTU per sample and the taxa with very low abundance might be overlooked^26^. Because only a fragment of 16 S rRNA gene is sequenced, the resolution may not invariably be adequate enough to species level. Given that it is difficult to design a universal PCR primer that amplifies the gene sequences from all the gut inhabitants with ‘equal efficiency’, it becomes indispensable to enumerate particular bacterial groups with specific and sensitive primer sets so as to obtain detailed information. To this end, our culture-independent RT-qPCR approach exploits thoroughly validated and highly specific primers and targets 16 S rRNA ‘molecules’ which are present in thousands to tens of thousands of copies per cell (approximately 10^3^ to 10^4^ molecules per actively growing cell; vs. only a few to a dozen copies of rRNA genes), thereby yielding a sensitive and precise quantification data^17, 18, 20, 22, 27^. Thus, our data fortifies the existing literature of gut microbiota with important numeric and longitudinal data about bifidobacterial population, particularly in context to infancy and early childhood.

The reports of bifidobacterial species composition during infancy have been somewhat divergent plausibly because of variations in cohort age, size and geographical location or methods used^26^. While many studies have suggested that bifidobacteria are the predominant constituent of neonatal gut microbiota^28–31^, other studies have reported high fluctuations in bifidobacterial abundance varying from very low abundance to absence in many subjects^2, 5, 11, 32–34^. Our data seem to concur with the latter opinion because we also noted that many infants may not carry any bifidobacterial species even until 1 or 3 months of age or maybe the carriage is too low to be technically perceived (Fig. 1A and Suppl. Fig. 1). Indeed, with a detection rate of more than 95%, bifidobacteria appeared to be a core microbiota element from M3 onwards. However, in terms of bacterial count (or in other words, bacterial abundance), bifidobacteria did not truly represent the most abundant clade when we estimated the bacterial predominance in these 76 infants by appraising the counts of different major gut bacterial groups and arbitrarily identifying the most abundant clade i.e., the one with highest bacterial count at a given time-point (Suppl. Fig. S2). In this estimation, we found that at first day of life, the microbiota was predominated mainly by facultative aerobes including Enterobacteriaceae and *Staphylococcus* or by *Bacteroides fragilis* group in some babies but this dominance gradually transitioned to strict anaerobes including *B. fragilis* group and bifidobacteria. Finally, by age 3 years, this predominance switched to a diverse spectrum of Clostridiales including *C. coccoides* group or *C. leptum* subgroup, possibly owing to the effect of weaning and/or transition towards strictly anaerobic adult-like microbiota configuration. Bifidobacterial predominance was detected only in 3% babies at D1, 25–35% babies until M1, 49–51% babies at M3 and M6 and 14% babies at Y3 (Suppl. Fig. S2). This pattern of bacterial succession is in line with previous reports^6, 7, 11, 35^ and might indicate that the bifidobacterial abundance could vary from very low to very high during infancy and early childhood. At age 3Y, the bifidobacterial microbiota was dominated by *B. longum* and *B. breve* followed by *B. catenulatum* group and *B. bifidum* (Fig. 1). However, in a previous study on healthy Japanese adults (*n* = 46; age: 37 ± 9 years), our colleagues reported that adult bifidobacterial microbiota is predominated mainly by *B. longum*, *B. catenulatum* group and *B. adolescentis* ^52^. This might indicate that the bifidobacterial population is still under transition state at age 3 years and stabilizes later on. Recently, our colleagues also reported that early life microbiota transitions could be shaped by the class of breast-milk oligosaccharides (e.g. fucosyllactose) as well as the genetic capacity of infant bifidobacterial subsets to utilize these ingredients^7^. Together, these studies clearly merit further exploration of the key factors that shape the neonatal *Bifidobacterium* development. For example, we also noted that boys had a higher *Bifidobacterium* count (*P* = 0.01) and prevalence (*P* = 0.07) at first day of life compared to girls (Suppl. Fig. S3); and the carriage of *B. longum* also remained on a higher side in boys during the first 6 months, with difference being statistically significant at 6 M (*P* = 0.03). Although the relationship between gender and microbiota remains to be established, some studies do have pondered upon this connection^35–37^ and hence research on this aspect should be worth exploring.

For decades, the intrauterine environment had been believed to be sterile. However, recent studies have demonstrated that the fetus may already be exposed to a variety of bacteria *in-utero* ^14, 39–41^. Bacteria frequently detected in prenatal niches mainly belong to enterobacteria, enterococci, staphylococci, streptococci and propionibacteria; however, DNA of bifidobacteria and lactobacilli have also rarely been detected^41^. Recently, we reported that the meconium of cesarean-born babies manifests remarkably lower carriage of lactobacilli compared to vaginally-born babies^16^. In the present study, we also detected one or more bifidobacterial species in the first-pass meconium samples of 21% of babies (Fig. 1). Like lactobacilli, bifidobacteria are also by and large believed to be transmitted vertically from mother to baby during vaginally delivery. However, we found an equivalent carriage of bifidobacteria in the meconium of VG and CS babies, hinting that some bifidobacterial clades may have colonized these infants before birth. Although, we also noted that, compared to VG babies, CS babies may experience relatively lower carriage of bifidobacteria during subsequent time-points until M3 (Fig. 4). This finding corroborates previous reports^5, 6, 14, 40, 42^ suggesting that the mother-to-baby transmission of bifidobacteria do occur in CS babies but the frequency and/ or spectrum of this transmission may be lower as compared with vaginal birth. Nevertheless, even though all the differences of birth mode tended to vanish by age 6 months or 3 years, these results together might hint that most of the bifidobacterial clades in newborn gut are inherited from the mother and the birth mode might influence their succession during first few months of life.

Birth mode and feeding type are undoubtedly the two most important determinants of the early life gut microbiota configuration. Following a heavy impact of birth mode, the microbiota development during subsequent weeks is guided by the mode of feeding^1, 5, 6, 11^. By and large, bifidobacteria are frequently found to dominate the microbiota of breast-fed infants worldwide, in some cases constituting up to 80–90% of the bacterial population^43, 44^. Mother’s milk provides an abundant source of growth factors including human milk oligosaccharides (N-acetyl-glucosamine, lactulose etc.) on which gut bifidobacteria thrive and thus confer multiple health benefits to the newborn, including improved gut barrier function and defense from enteric pathogens^7, 45^. In addition, breast-milk is also suggested to routinely harbor several bifidobacterial taxa, mainly *B. longum*, *B. breve* and *B. bifidum* ^45, 46^. However, we found that the count of all major bifidobacterial members during the first 6 months remained considerably higher in mixed-fed infants as compared to exclusively breast-fed babies (Fig. 2). Notably, due to paucity of detailed information, we could not identify exclusively formula-fed babies and hence we considered all cases of formula-feed exposure as mixed-feeding. As mentioned previously, most infant-formula supplements presently prevalent in Japan contain prebiotic compounds such as galacto-oligosaccharides, fructo-oligosaccharides, etc. (concentration varying from 0.3 to 2.3% depending on the brand) that are known to augment the growth of bifidobacteria^47, 48^. In our study, about 77% of mixed-fed infants were confirmed to be taking formula-feed fortified with galacto- or fructo-oligosaccharides, lactulose, or raffinose. Notably, among these babies, about 90% were taking a galacto-oligosaccharide fortified (concentration: ≈1.2%) infant formula. Regarding other babies, we could not retrieve detailed information of formula milk consumed. Nevertheless, our data concurs with recent reports^40, 48, 49^ suggesting that prebiotic-enriched formula may shift the microbiota of breast-fed babies towards a bifidobacteria-enriched configuration.

The positive correlation of bifidobacteria with other strict anaerobes and a negative correlation with facultative anaerobes at one or more time-points during the first 6 month (Fig. 3) concurs with the patterns of gut bacterial colonization (Suppl. Figs S1 and S2), indicating a transition from an abundance of facultative anaerobes towards the dominance of strict anaerobes. Notably, the prominent correlation of bifidobacteria with Enterobacteriaceae (negative) and acetate (positive) also coincides with birth mode and feeding type analyses wherein we found that besides having higher carriage of bifidobacteria, VG as well as MF infants also had higher fecal acetate concentration (Suppl. Fig. S4). These data might corroborate that bifidobacteria antagonize Enterobacteriaceae and *Enterococcus* apparently by producing acetate and thus might also protect the neonate from enteropathogenic infections^7, 50^. Intriguingly, bifidobacteria shared a negative correlation with succinate (Fig. 3). Not much is known about the role of succinate in the gut. Being an intermediate of bacterial carbohydrate metabolism, succinate does ─ or should ─ not typically accumulate to a substantial extent in the bowel of healthy humans. Interestingly, succinate is increased with high-fat diets^51^ and, if accumulated in excess, may have negative effect on colonic mucosa^53^ and immune/ inflammatory health^54^. Thus, the negative correlation of bifidobacteria with succinate might indicate some sort of host-beneficial or -symbiotic attribute. Perhaps, bifidobacteria control succinate by checking the growth of succinate-producing facultative anaerobes viz. enterobacteria, enterococci etc., or maybe this relationship is dependent upon the positive correlation of bifidobacteria with clades that convert succinate to glucose, propionate etc. Nevertheless, the exact reason remains unclear and should be an interesting topic for future studies.

Numerous studies have examined the *Bifidobacterium* carriage in infants; however, the data at species level remains limited and disparate due to different analytical methods or due to differences in cohort size, age, genetic and geographical backgrounds etc. Traditionally, *B. infantis* has been considered as one of the most prevalent bifidobacterial species during infancy^55–58^ but we did not find such predominance of *B. infantis*. On the contrary, we found *B. longum* and *B. breve* as the predominant bifidobacterial species. Studies on infants from Finland^59^, Ireland^3^, Brazil^60^, Sweden^5^ and Belgium^40^ have reported similar predominance of *B. longum*, thereby suggesting its vast ability to adapt to the human intestinal environment while also corroborating its prominent vertical transmission. However, the dominance of *B. breve* in this Japanese cohort discords from other studies and could be attributed to two reasons specific to Japanese population: (a) the prevalence of oligosaccharides-supplemented formula-feed since *B. breve* is well-known to grow well on such prebiotics substrates^61^, and (b) the high prevalence and consumption of probiotic products containing *B. breve* strains. In addition, both these factors might also describe the low prevalence of *B. infantis* in Japanese babies because *B. infantis* is a prodigious consumer of human-milk oligosaccharides but can easily be outnumbered by other species such as *B. longum* and *B. breve* if the environment is rich in plant-derived oligosaccharides^62^.

The present study had several limitations most important of which was our inability to assess the influence of factors such as maternal diet, antibiotic exposure, siblings etc. because of the paucity of information. Also, the effect of maternal (fecal, vaginal, skin etc.) microbiome was not examined because maternal samples were not collected. The large difference between the numbers of vaginally- vs. cesarean-born babies may also be a limitation in this observational study. Although we herein examined the eight most-frequent and most-predominant infant-related bifidobacterial species, our data however might not truly represent the whole bifidobacterial community since several previous studies have also suggested the potential presence of other (or unknown) bifidobacteria in the infant gut^3, 5, 31, 59, 60^. Nevertheless, all infants as well as mothers enrolled in the study were apparently healthy and hence the results could be extrapolated to general population. In summary, our study of a large unselected cohort of healthy term infants demonstrate a quantitative bird’s-eye view of age-related succession of bifidobacterial community during the critical developmental stages of life. We also show that *B. longum* subsp*. longum* and *B. breve* are probably the most central bifidobacterial clades in the Japanese infants’ gut. It should be an interesting subject for future studies to compare these colonization patterns with cohorts from other geographical regions. At the same time, it remains imperative to elucidate the routes and sources of bifidobacteria in the neonatal gut as well as to decipher the roles of specific bifidobacterial clades in the infant gut. Taken together, our quantitative and longitudinal data should prove to be informative and facilitative for prospective studies investigating diverse aspects of bifidobacteria in particular context to the early life gut microbiota in health and disease.

## Methods

### Study subjects

The study included 76 healthy full-term vaginally-born Japanese infants enrolled at the Gonohashi Obstetrics and Gynecology Hospital, Tokyo. The infants were part of a large cohort of more than 150 Japanese infants wherein fecal carriage of various gut microbes during the first three years of life has previously been studied^16, 23^. However, analysis of *Bifidobacterium* subgroups and species was not previously examined. Therefore, we herein investigated the progression of bifidobacterial community in a selected group of 76 vaginally-born infants whose samples were accessible at all the time-points from 1 day to 3 years of age. The general information about these infants is provided in Table 1, and the detailed information about the cohort can be found elsewhere^23^. For comparison purposes, the data of these 76 infants was also compared with a counterpart group of 13 babies that were delivered via elective C-section. The general information about these infants is provided in Suppl. Table S1. All the infants as well as their mothers remained apparently healthy with no indication of any major illness during the study period.

**Table 1:** General characteristics of the infants enrolled in the study including birth mode, gender, weight, antibiotics, and feeding mode.

Characteristics	Age					
	1 day	7 days	1 month	3 months	6 months	3 years
Total number of infants	76	−				
Boy: Girl	40: 36	−				
Days (average) spent in hospital post-birth	4.1 ± 0.5	−				
Bodyweight^*^	2.9 ± 0.4^§^	2.9 ± 0.4	3.7 ± 0.5	5.7 ± 0.7	7.2 ± 0.7	13.2 ± 1.3
Exclusively breast-fed	76	27	22	29	12	−
Mixed-fed	0	49	54	47	64	−
First exposure to formula-feed	0	49	5	4	10	−
Antibiotic exposure	1	0	0	0	1	0

^*^Mean ± SD, Kilograms. ^§^Birth-weight.

### Ethical statement

The study protocol was approved by the ethical committees of Juntendo University and Yakult Central Institute. In accordance with the Declaration of Helsinki, prior written informed consent was obtained from all the parents or legal representatives. All methods and experiments were performed in accordance with the relevant guidelines and regulations.

### Sample collection

The fecal samples were collected at six time-points i.e., 1 day (D1), 7 days (D7), 1 month (M1), 3 months (M3), 6 months (M6) and 3 years (Y3) of age. A spoonful (0.5 to 1.0 g) of fecal sample was collected fresh in a fecal collection tube (Sarstedt AG & Co., Numbrecht, Germany) containing 2 ml of RNA*later*, an RNA stabilizing reagent (Ambion, Austin, TX); another spoonful was collected into an empty tube for organic acids analysis. All D1 samples were from the first intestinal discharge i.e., first-pass meconium (obtained from first diaper) of which 74 (97.4%) were passed within 24 h after birth whereas 2 samples were discharged between 24 and 48 h. Immediately after collection, samples were stored in the refrigerator (3–4 °C) anaerobically by using Anaero Pouch-Anaero (Mitsubishi Gas Chemical Company, Inc., Tokyo, Japan) and were sent immediately in a cooling box with refrigerants and anaero-packs to the research lab where these were stored at 3–4 °C in a Biosafety Category II microbiology laboratory until further processing.

### Sample processing and RNA extraction

Prior to nucleic acid extraction, the samples were subjected to a pretreatment step as follows: the sample was weighed and suspended in 9 volumes of RNA*later* to make a fecal homogenate (100 mg feces/ ml). One ml of PBS(-) was added to 200 µl of this fecal homogenate following which the suspension was centrifuged at 13,000 g (4 °C) for 10 min, the supernatant was discarded, and the precipitating pellet was stored at −80 °C until nucleic acids extraction. RNA extraction were done by using previously described methods^18^. Briefly, the thawed sample was re-suspended in a solution containing 346.5 µl of RLT buffer (Qiagen Sciences, Germantown, MD), 3.5 µl of β-mercaptoethanol (Sigma-Aldrich Co., St. Louis, MO), and 100 µl of Tris-EDTA buffer. Glass beads (BioSpec Products, Inc., Bartlesville, OK) (300 mg; diameter, 0.1 mm) were added to the suspension, and the mixture was subjected to a vigorous vortex procedure for 5 min using a ShakeMaster Auto apparatus (Bio Medical Science Inc., Tokyo, Japan). Acid phenol (Wako Pure Chemical Industries, Ltd., Osaka, Japan) (500 µl) was added, and the mixture was incubated for 10 min at 60 °C. After phenol-chloroform purification and isopropanol precipitation, the nucleic acid fraction was suspended in 0.2 ml of nuclease-free water (Ambion, Inc.).

### RT-qPCR and bacterial quantification

Fecal counts of *Bifidobacterium* groups/ species were analyzed by using a sensitive quantitative analytical system based on RT-qPCR targeting 16 S rRNA molecules, as per the methods described previously^17, 18, 20, 22, 27^. Briefly, RT-qPCR analysis was executed with a Qiagen OneStep RT-PCR kit (Qiagen GmbH, Hilden, Germany). Each reaction mixture (10 µl) comprised 1X Qiagen OneStep RT-PCR buffer, 0.5X Q-solution buffer, each deoxynucleoside triphosphate at a concentration of 400 µM, a 1:100,000 dilution of SYBR green I (BioWhittaker Molecular Applications, Rockland, ME), 0.4 µl of Qiagen OneStep RT-PCR enzyme mixture, and 5 µl of template RNA. Each primer pair was added at a concentration of 0.6 µM. For reverse transcription, the reaction mixture was incubated at 50 °C for 30 min. The subsequent continuous amplification program consisted of one cycle at 95 °C for 15 min, followed by 40 cycles at 94 °C for 20 s, 55 °C (60 °C for *B. infantis*) for 20 s, and 72 °C for 50 s. The fluorescent products were detected in the last step of each cycle. A melting curve analysis was performed after amplification to distinguish the targeted PCR products from the non-targeted ones. The melting curve was obtained by slow heating at temperatures from 60 to 95 °C at a rate of 0.2 °C/s with continuous fluorescence collection. Amplification and detection were performed in 384-well optical plates with an ABI PRISM^®^ 7900HT sequence detection system (Applied Biosystems, Foster, CA). Standard curves for the corresponding standard bacterial strains were generated by using C_q_ (quantification cycle) values and the corresponding cell counts, which were determined microscopically with the DAPI (4′,6-diamidino-2-phenylindole) staining method as previously described^18^. To determine the target bacterial populations in the fecal samples, 1/20,000, 1/200,000, and 1/2,000,000 portions of the RNA solution were subjected to RT-qPCR. The C_q_ values in the linear range of the assay were applied to the analytical curve generated in the same experiment to obtain the corresponding bacterial count in each nucleic acid sample; this count was then converted to the count per gram of fecal sample. The details of methodological validation of these *Bifidobacterium* group- and species-specific assays have been described elsewhere^27, 56^. All the primers have been thoroughly validated with RNA fractions extracted from various target and non-target reference bacterial strains^27, 56^. Briefly, the specificity of each primer set was confirmed against RNA fractions extracted from 10^5^ cells of each bacterial strain by using RT-qPCR. The amplified signal was considered positive (+) at >10^4^ standard cells, positive/negative (±) at 10^4^ to 10° standard cells, and negative (−) at <10° standard cells. The amplified signal was also defined as negative (−) when the corresponding melting curve had a peak different from that of the standard strain. Detection sensitivity was evaluated by using RNA fractions extracted from culture samples of corresponding reference strain in the early stationary phase (24 h), wherein bacterial counts were determined microscopically by DAPI staining. Serial RNA dilutions corresponding to bacterial counts ranging from 10^−3^ to 10^5^ cells were assessed by RT-qPCR assay. The range of RNA concentrations at which there was linearity with C_q_ value was confirmed (*R* ^2^ > 0.99). The minimum detection limit of these assays was 10^4^ cells/ g feces. The details of these primers’ sequences and corresponding annealing temperatures have been provided in the supplementary material (Supplementary Table S2). The count of genus *Bifidobacterium* (or total bifidobacterial count) was estimated as the sum of the counts of *B. catenulatum* group, *B. longum* subsp*. longum*, *B. longum* subsp*. infantis, B. bifidum, B. breve, B. adolescentis, B. angulatum*, and *B. dentium*. *B. catenulatum* group includes *B. catenulatum* and *B. pseudocatenulatum*. The counts of other bacterial groups including *Clostridium coccoides* group, *Clostridium leptum* subgroup, *Bacteroides fragilis* group, *Atopobium* cluster, *Prevotella*, *Lactobacillus*, Enterobacteriaceae, *Enterococcus*, *Staphylococcus, Streptococcus*, and *C. perfringens* were previously analyzed by 16 S or 23 S rRNA molecules-targeted RT-qPCR assays, as described previously^23^.

### Measurement of fecal organic acids

Fecal concentrations of organic acids viz. acetate, lactate, succinate, propionate, formate, butyrate, isobutyrate, valerate and isovalerate were measured by an in-house standard HPLC method using a Waters system (Waters 432 Conductivity Detector; Waters Co., Milford, MA) equipped with two columns (Shodex RSpack KC-811, column size 8 × 300 mm, Showa Denko Co. Ltd., Tokyo, Japan), as per the method described previously^23^. The concentration of each organic acid was normalized against that of the corresponding external standard. The total organic acids concentration was estimated as the sum of the fecal concentration of all of the abovementioned organic acids.

### Statistical analyses

The results of bacterial count (log_10_ cells per gram of feces) are expressed as mean ± standard deviation. The carriage rate or prevalence of colonization was expressed as the percentage of infants in which the specific bacterium was detected. The differences in bacterial counts by delivery mode, feeding type and gender were calculated by two-tailed unpaired Student’s *t*-test; prevalence was compared by using Fisher’s exact probability test. Hierarchical clustering and heat-maps depicting the patterns of bifidobacterial colonization were constructed in R software (version 3.3.2) using the ‘heatmap.2’ package. Scattered dot-plots were generated in GraphPad Prism 6.0 software system. Correlation of fecal count of bifidobacterial clades with that of other gut bacteria and with fecal concentration (µmol/g feces) of different organic acids was estimated by using Spearman’s rank correlation coefficient test (GraphPad Prism software system, version 6.0). Unless otherwise stated, a value of *P* < 0.05 was considered statistically significant.

### Data availability

All data generated or analyzed during this study are included in this published article (and its Supplementary Information files). Other information/ data related to the current study are available from the corresponding author on reasonable request.

## Electronic supplementary material

Supplementary material.
