# Supplementary material for: Evolution of gut Bifidobacterium population in healthy Japanese infants over the first three years of life: a quantitative assessment
Source: Sci Rep. 2017 Aug 30;7:10097. doi: 10.1038/s41598-017-10711-5 (PMC5577255; doi:10.1038/s41598-017-10711-5)
Supplement: Supplementary file 1 — Supplementary material. [file 41598_2017_10711_MOESM1_ESM.pdf]

Evolution of gut *Bifidobacterium* population in healthy Japanese infants over the first three years of life: a quantitative assessment

Ravinder Nagpal[1¶*], Takashi Kurakawa[2], Hirokazu Tsuji[2], Takuya Takahashi[2], Kazunari Kawashima[3], Satoru Nagata[4], Koji Nomoto[2], Yuichiro Yamashiro[1]

[1]Probiotics Research Laboratory, Juntendo University Graduate School of Medicine, Hongo 2-9-8-3F, Bunkyo-ku, Tokyo 113-0033, Japan.

[2]Yakult Central Institute, Kunitachi-shi, Tokyo, Japan.

[3]Gonohashi Obstetrics and Gynecology Hospital, Koto-ku, Tokyo, Japan.

[4]Department of Pediatrics, School of Medicine, Tokyo Women's Medical University, Tokyo, Japan.

[¶]Present affiliation: Gut Microbiome and Metabolic Diseases, Wake Forest Biotech Place, Wake Forest School of Medicine, Winston-Salem, NC 27101, USA.

***Address correspondence to: ravinder@juntendo.ac.jp

SUPPL. TABLE S1. General characteristics of the cesarean-born infants enrolled in the study.

Characteristics	Age					
	1 day	7 days	1 month	3 months	6 months	3 years
Total number of infants	13	–				
Boy : Girl	9 : 4	–				
Days (average) spent in hospital post-birth	6.7±1.6	–				
Bodyweight*	3.0±0.3§	3.0±0.4	3.8±0.4	5.6±0.5	7.3±0.6	13.9±1.7
Exclusively breast-fed	13	4	4	6	4	–
Mixed-fed	0	9	9	7	9	–
First exposure to formula-feed	0	9	0	0	1	–
Antibiotic exposure	0	0	0	0	0	0

*Mean ± SD, Kilograms. §Birth-weight.

SUPPL. FIG. S1. Varied patterns in the infant gut *Bifidobacterium* colonization. Clustered heat-map depicting the arrays of fecal carriage of genus *Bifidobacterium* viewed over the first 3 years of life in healthy full-term Japanese infants (n=89) born vaginally (n=76) or by C-section (n=13). The count of genus *Bifidobacterium* was expressed as the sum of the fecal counts (mean±SD; $\log_{10}$ cells/g feces) of *B. longum* subsp. *longum*, *B. longum* subsp. *infantis*, *B. breve*, *B. catenulatum* group, *B. bifidum*, *B. adolescentis*, *B. angulatum* and *B. dentium*. *B. catenulatum* group includes *B. catenulatum* and *B. pseudocatenulatum*. Age (*x*-axis): 1 day, 7 days, 1 month, 3 months, 6 months, 3 years.

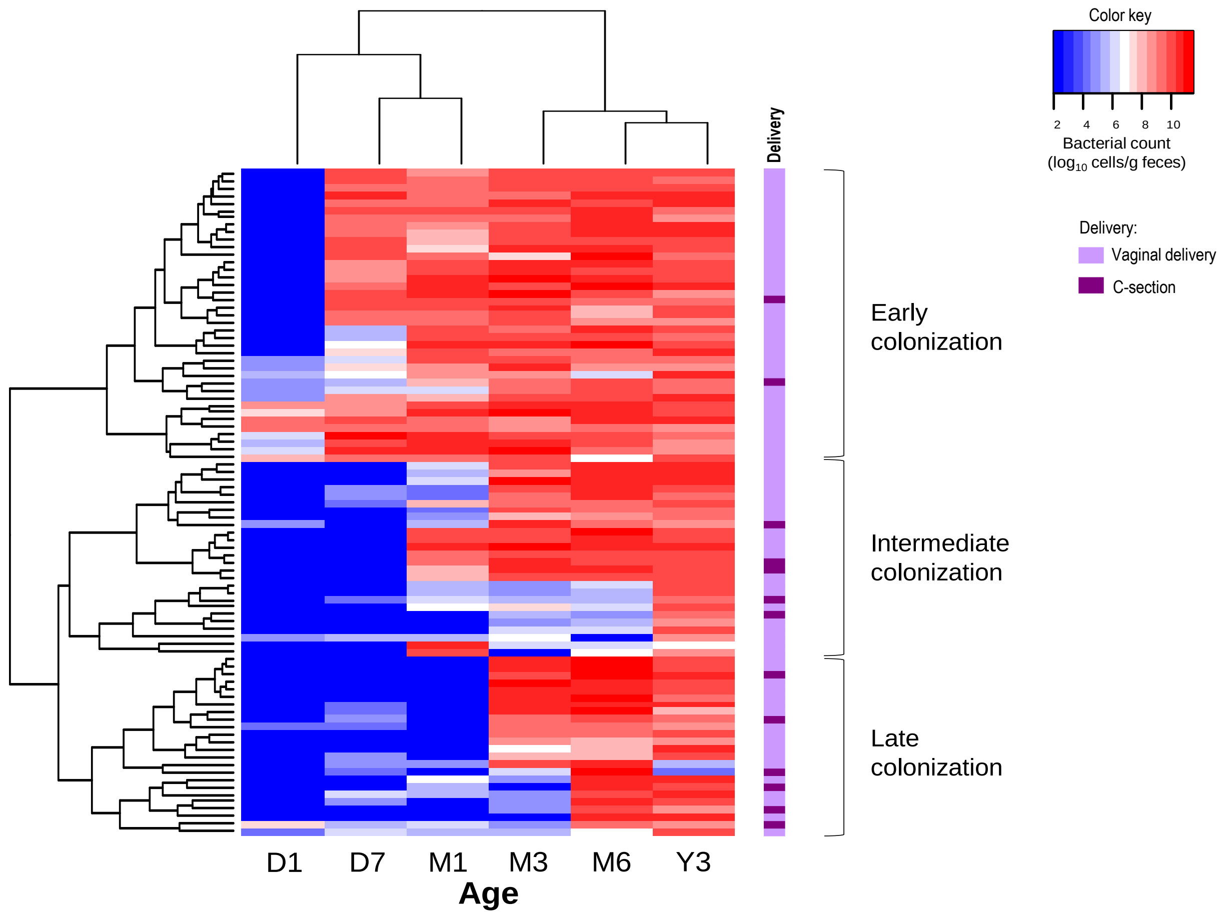

SUPPL. FIG. S2. An arbitrary overview of the bacterial abundance/ predominance in healthy full-term vaginally-born infants (n=76) during the first 3 years of life. The abundance was estimated based on the fecal bacterial counts (log_{10} cells/g feces) wherein the bacterial clade with highest fecal count at a given time-point was considered as most abundant clade among all the targeted bacterial groups. Age (*x*-axis): 1 day, 7 days, 1 month, 3 months, 6 months, 3 years.

Dominant clade

D1	D7	M1	3M	6M	Y3
BC	BC	BC	BC	EB	BF
BL	ST	BL	BB	EB	CC
ST	BL	BL	BL	BL	BC
EC	BL	BC	BC	BL	BF
EB	BB	BB	BB	CC	CL
ST	BB	BB	BB	CC	CL
EB	BL	BL	BB	BF	CL
EB	BL	BL	BL	BF	CL
LA	BL	BL	CC	BB	CL
ST	BB	BB	BF	BL	BF
BF	BL	BL	EB	BL	BF
BF	BB	BB	BF	BF	CL
EB	BL	BL	BF	BF	CL
EB	BL	EB	BL	BL	CL
EB	BL	AT	BB	BF	CL
BF	BB	BF	BB	BF	CL
EB	BB	EB	BF	BF	CL
EB	BB	BF	BF	BF	CL
EB	BB	BF	BF	EB	CL
ST	BB	BF	BF	BF	BL
EB	EB	BB	BB	BB	BF
EC	EB	BB	BB	BB	CC
BF	EC	BB	BB	BB	CL
	EB	BC	BC	BL	BF
LA	EB	BL	BL	BL	CC
EB	EC	BL	BL	BL	CC
EB	BF	BC	BB	BB	CL
EB	EB	BL	BC	BB	BF
BF	BF	BC	BC	BC	BF
AT	BF	BC	BC	BF	CL
EB	BF	BL	BF	BF	CL
EB	EB	BL	BF	BF	BL
BF	EB	BL	CC	CC	CC
ST	EB	EB	BIF	BB	BC
EB	EC	BF	BC	BC	BC
BF	EC	EB	BB	BB	BL
EB	BF	BF	BB	BB	BF
BF	EB	BF	BB	BB	BF
BF	EB	EB	BB	BB	CC
BF	BF	BF	BB	BB	CL
EB	ST	BF	BB	BIF	CL
ST	CP	EB	BB	BB	CL
EB	EB	BF	BC	BC	BF
EC	EB	EB	BB	BB	CL
ST	EC	EB	BL	BB	CL
ST	BF	EB	BB	BL	CL
EB	EB	EB	BL	BL	BF
EB	EB	BF	BB	BF	BF
CL	EB	BF	BB	CC	CC
ST	EC	BF	BIF	EB	CL
EC	BF	BF	BL	EB	CL
LA	EB	CC	EB	BIF	BC
EB	BF	EB	BF	BD	BL
CL	EC	EB	EB	BL	CC
BF	BF	BF	BF	BL	CL
BF	BF	EB	BF	BL	CL
EB	EB	EB	EB	BIF	CL
EB	BF	EB	BF	BB	CL
ST	EB	EB	EB	BB	CL
BF	CC	CC	CC	AT	BC
ST	EB	EB	EB	EB	BC
BF	EB	EB	LC	BF	BB
BF	EB	EB	EB	EB	AT
ST	BF	BF	BF	BF	BF
BF	EC	EB	EC	EB	BF
EB	BF	CC	EB	BF	CC
BF	BF	BF	BF	BF	CL
ST	BF	BF	BF	BF	CL
EB	CC	BF	BF	BF	CL
BF	EB	EB	EB	BF	CL
CC	EB	EB	CC	CC	CL
BF	BF	EB	BF	CL	CL
EB	EB	EB	EB	EB	CL
EB	EB	EB	EB	EB	CL
ST	EB	EB	EB	EB	CL
EB	EB	EB	BF	EB	PR

Color code

BL	*B. longum*
BB	*B. breve*
BIF	*B. bifidum*
BC	*B. catenulatum* gp.
CC	*C. coccoides* gp.
CL	*C. leptum* subgp.
BF	*B. fragilis* gp.
AT	*Atopobium* cluster
PR	*Prevotella*
CP	*C. perfringens*
EB	Enterobacteriaceae
EC	*Enterococcus*
ST	*Staphylococcus*
LA	*L. acidophilus*
LC	*L. casei*

SUPPL. FIG. S3. Count and prevalence of bifidobacteria in vaginally-born babies (n=76) profiled by gender (boys: *n*=40; girls: *n*=36). Bacterial count is expressed as mean ± standard deviation of $\log_{10}$ cells/g feces. Prevalence (detection rate, %) is expressed as the percentage of infants in which the specific bacterium was detected. The count of genus *Bifidobacterium* represents the sum of the counts of *B. longum* subsp. *longum*, *B. longum* subsp. *infantis*, *B. breve*, *B. catenulatum* group, *B. bifidum*, *B. adolescentis*, *B. angulatum* and *B. dentium*. *B. catenulatum* group includes *B. catenulatum* and *B. pseudocatenulatum*. Age (*x*-axis): 1 day, 7 days, 1 month, 3 months, 6 months, 3 years. *P<0.05, **P<0.01 (count: Student's *t*-test; prevalence: Fisher's exact probability test). Only bacteria with notable difference are shown here. Age (*x*-axis): 1 day, 7 days, 1 month, 3 months, 6 months, 3 years.

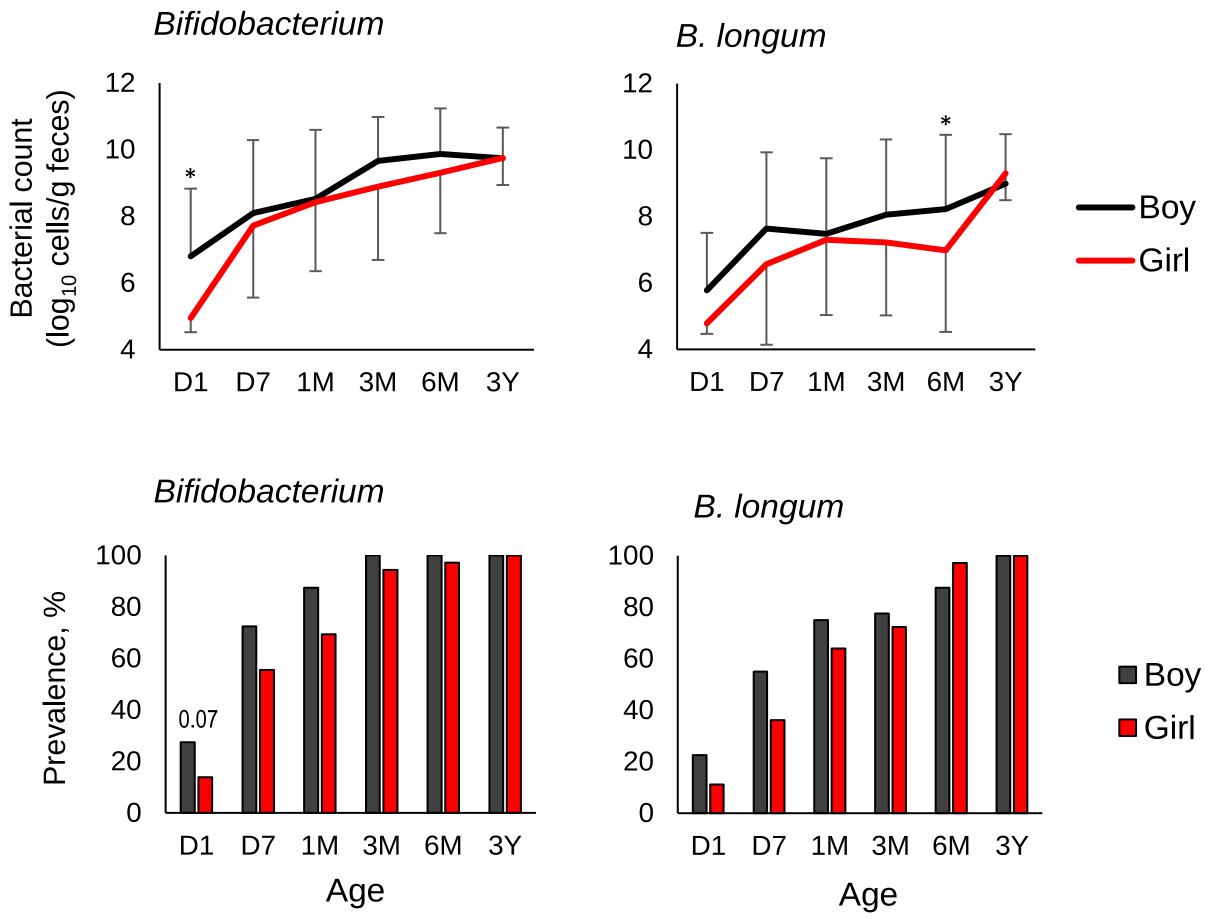

SUPPL. FIG. S4. Fecal organic acid concentration (μmol/g feces) as a function of delivery mode (A) or feeding type (B). *P<0.05, **P<0.01 (Student's *t*-test). Only organic acids with notable difference are shown here. Age (*x*-axis): 1 day, 7 days, 1 month, 3 months, 6 months, 3 years.

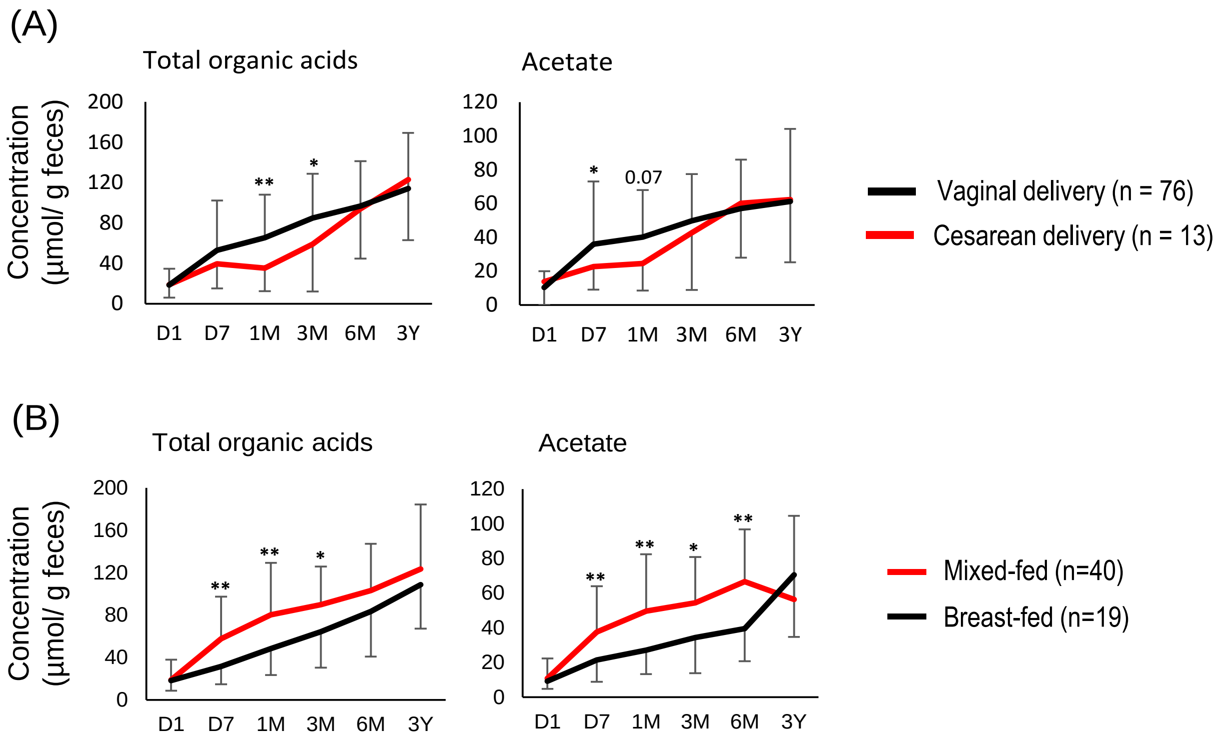

SUPPL. Table S2. List of targeted bifidobacterial groups/ species and the corresponding target gene, primer sequence, annealing temperature and product size.

Target bacterium	Target rRNA gene	Primer	Sequence (5'-3')	Annealing temperature (°C)	Amplicon size (bp)	Reference
B. longum subsp. *longum*	23S	BLON-23S-F<br>BLON-23S-R	TGGGACAGGCAAATCCGTC<br>TTTGAGCCTGACGCCGAAGC	55	110	[1]
B. longum subsp. *infantis*	23S	BINF-23S-F<br>BINF-23S-R	GTCCCGTAGGCAAATCCGC<br>AAAAGCTCCCCGCGCGAGGC	60	261	[1]
B. breve	16S	BiBRE-1<br>BiBRE-2	CCGGATGCTCCATCACAC<br>ACAAAGTGCCTTGCTCCCT	55	288	[2]
B. bifidum	16S	BiBIF-1<br>BiBIF-2	CCACATGATCGCATGTGATTG<br>CCGAAGGCTTGCTCCCAAA	55	278	[2]
B. catenulatum group*	16S	BiCATg-1<br>BiCATg-2	CGGATGCTCCGACTCCT<br>CGAAGGCTTGCTCCCGAT	55	285	[2]
B. adolescentis	23S	BADO-23S-F<br>BADO-23S-R	AGCAATCTTCATGGTTGC<br>ACCGTCTCGGTTTTGCCGGTCCATG	55	269	[1]
B. angulatum	16S	BANG-F<br>BANG-R	TGCTCCAGTCCATCGC<br>TTACCCCGCCGACTACC	55	275	[1]
B. dentium	16S	BiDEN-1<br>BiDEN-2	ATCCCGGGGGTTCGCCT<br>GAAGGGCTTGCTCCCGA	55	387	[2]

**B. catenulatum* group includes *B. catenulatum* and *B. pseudocatenulatum*.

[1]. Kurakawa, T. *et al*. Establishment of a sensitive system for analysis of human vaginal microbiota on the basis of rRNA-targeted reverse transcription-quantitative PCR. *J. Microbiol. Methods* **111**, 93-104 (2015b).

[2]. Matsuki, T., Watanabe, K., Tanaka, R., Fukuda, M. & Oyaizu, H. Distribution of bifidobacterial species in human intestinal microflora examined with 16S rRNA-gene-targeted species-specific primers. *Appl. Environ. Microbiol.* **65**, 4506-4512 (1999).
